# Computational Exploration of Minimum Energy Reaction Pathway of N_2_O Formation from Intermediate *I* of P450nor Using an Active Center Model

**DOI:** 10.3390/ijms242417172

**Published:** 2023-12-06

**Authors:** Yusuke Kanematsu, Hiroko X. Kondo, Yu Takano

**Affiliations:** 1 Graduate School of Advanced Science and Engineering, Hiroshima University, 1-4-1 Kagamiyama, Higashi-Hiroshima 739-8527, Japan; 2 Graduate School of Information Sciences, Hiroshima City University, 3-4-1 Ozukahigashi Asaminamiku, Hiroshima 731-3194, Japan; h_kondo@mail.kitami-it.ac.jp; 3 Faculty of Engineering, Kitami Institute of Technology, 165 Koen-cho, Kitami 090-8507, Japan

**Keywords:** nitric oxide reductase, density functional theory, database analysis, local outlier factor, heme proteins, P450nor

## Abstract

P450nor is a heme-containing enzyme that catalyzes the conversion of nitric oxide (NO) to nitrous oxide (N_2_O). Its catalytic mechanism has attracted attention in chemistry, biology, and environmental engineering. The catalytic cycle of P450nor is proposed to consist of three major steps. The reaction mechanism for the last step, N_2_O generation, remains unknown. In this study, the reaction pathway of the N_2_O generation from the intermediate *I* was explored with the B3LYP calculations using an active center model after the examination of the validity of the model. In the validation, we compared the heme distortions between P450nor and other oxidoreductases, suggesting a small effect of protein environment on the N_2_O generation reaction in P450nor. We then evaluated the electrostatic environment effect of P450nor on the hydride affinity to the active site with quantum mechanics/molecular mechanics (QM/MM) calculations, confirming that the affinity was unchanged with or without the protein environment. The active center model for P450nor showed that the N_2_O generation process in the enzymatic reaction undergoes a reasonable barrier height without protein environment. Consequently, our findings strongly suggest that the N_2_O generation reaction from the intermediate *I* depends sorely on the intrinsic reactivity of the heme cofactor bound on cysteine residue.

## 1. Introduction

Denitrification is a type of anaerobic respiration performed by soil microorganisms. It is a biological process that uses nitrogen oxides as electron acceptors in the respiratory system to convert them to molecular nitrogen as follows: nitrate ion (NO_3_^−^) → nitrite ion (NO_2_^−^) → nitric oxide (NO) → nitrous oxide (N_2_O) → nitrogen molecules (N_2_) [1]. In denitrification, NO causes physiological reactions such as vasodilation [2], but is highly reactive, resulting in cytotoxicity through reactions with proteins and other biomolecules [3,4,5,6,7]. It is necessary to convert NO immediately in order to avoid such damage to the organism. Nitric oxide reductases (NORs) are responsible for the chemical conversion of NO to N_2_O by catalyzing a two-electron reduction reaction of NO. N_2_O is about 310 times more effective greenhouse gas than carbon dioxide and an ozone-depleting gas [8]. Since more than 75% of N_2_O in the global environment is produced by microorganisms such as molds and bacteria in soil, it is a fascinating issue not only in chemistry and biology but also in Wenvironmental engineering [9]. However, the molecular mechanism of N_2_O generation by NOR has not been fully solved for decades.

In the 1990s, there was a series of reports on isolating membrane-bound NORs from several denitrifying bacteria [10,11] and water-soluble NORs from denitrifying molds [12,13]. Both NORs contain heme iron as the active center. However, the structures and properties of the two enzymes are quite different, and the catalytic mechanisms of NO reduction reactions were expected to be very different. The crystal structure of membrane-bound NOR from *Pseudomonas aeruginosa* has recently been determined [14], and spectroscopic and computational studies have proposed its catalytic mechanism [15,16]. Computational study indicated that the N–N bond proceeds without an activation barrier but that there is an activation barrier of 20.9 kcal mol^–1^ for N–O bond cleavage via a hyponitrous intermediate [16].

**Figure 1:**
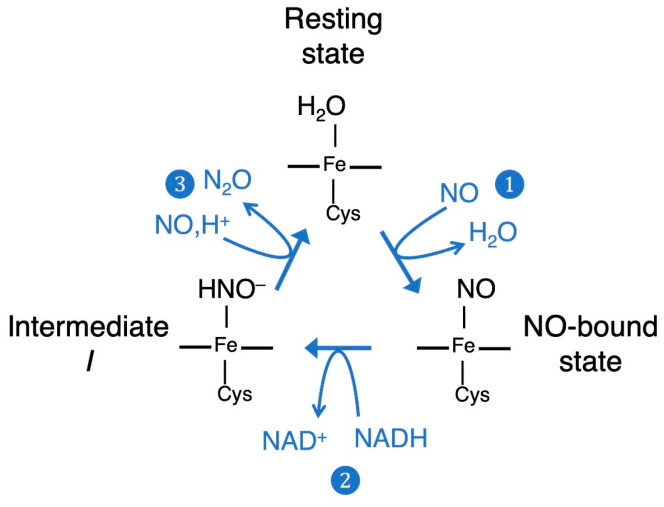
Catalytic cycle of P450nor that consists of (1) first NO binding, (2) hydride reduction using NADH yielding the intermediate *I*, and (3) second NO attack to generate N_2_O.

Water-soluble NOR isolated from the fungus *Fusarium oxysporum* shows approximately 40% sequence homology with cytochrome P450 [17] and is classified in the P450 superfamily, and thus named cytochrome P450nor [12,18,19]. Therefore, unlike other P450s involved in the metabolism of many organic molecules as a molecular oxygen monooxygenase [20], P450nor is unique in that it reduces NO molecules. From spectroscopic and X-ray crystallographic studies, the active center of P450nor is composed of heme *b* with the thiolate anion of Cys coordinated as the axial ligand of ferric iron in heme [18]. This enzyme catalyzes the reduction of NO to N_2_O with high efficiency using NADH as a cofactor, with a high turnover frequency [13,21,22], using the following chemical reaction: 2NO + NADH + H^+^ → N_2_O + NAD^+^ + H_2_O. This reaction is proposed to occur via three major chemical steps, as shown in Figure 1 [13]. First, NO is bound to the active center of the enzyme in a resting state, followed by the reaction with NADH to form intermediate *I*. Then, a second NO molecule attacks intermediate *I* to produce an N_2_O molecule.

In the resting state, the heme-thiolate active center has a low-spin ferric state with a water molecule as an axial ligand. First, the water molecule of the axial ligand is displaced from its active center, and NO is bound to it, producing an Fe-NO bonding center. Recently, time-resolved femtosecond crystallography using an X-ray free electron laser (XFEL) and quantum mechanics/molecular mechanics (QM/MM) calculations have enabled the successful characterization of the coordination and electronic structure of the Fe^3+^-NO complex in the absence of X-ray damage [23]. The determined NO-bound state has a short Fe-NO bond (1.67 Å) and a slightly bent NO coordination structure (Fe-N-O ≈ 158°). Subsequently, the NO-bound active center undergoes a direct two-electron reduction from NADH, forming the intermediate *I*. Although numerous studies have been performed using spectroscopy, theoretical calculations, and synthetic complex models [23,24,25,26,27], the structure of intermediate *I* was not determined due to its short lifetime. However, coupling studies of time-resolved spectroscopy, time-resolved crystallography using XFEL, and computational chemistry have recently revealed that the coordination and electronic structure of the key intermediate *I*, is the Fe^3+^-NHO^•−^ species [28].

In the last step, the intermediate *I* reacts with a second NO molecule, leading to the formation of N_2_O. Time-resolved Raman spectroscopy suggests a transient formation of a hyponitrous intermediate, ONNHO^−^, by a second NO attack [29]. Although there was a theoretical study using a QM/MM method [26,27], it was based on the Fe^3+^-NHOH^•^ species. The detailed reaction pathway for N_2_O formation beginning with the recently identified Fe^3+^-NHO^•−^ species has not been clarified. In this study, we carried out density functional theory (DFT) calculations using the active center model as the first step to clarify the molecular mechanism of the reaction of the identified intermediate *I* (Fe^3+^-NHO^•−^ species) with the second NO to form N_2_O. At first, the validity of the active center model was evaluated based on a database analysis (steric effect of protein environment) and DFT calculations (electrostatic effect of protein environment). In the validation based on the database analysis, we statistically analyzed the heme distortions sterically caused by the protein environment among oxidoreductases including the P450 superfamily using PyDISH [30] (https://pydish.bio.info.hiroshima-cu.ac.jp/, accessed on 18 October 2023) and local outlier factors (LOFs). The analysis revealed P450nor shares a common heme distortion with other P450 families, suggesting that the steric protein environment of P450nor would not adequately explain its unique property of converting of intermediate *I* to N_2_O. In the theoretical calculations, the binding energy of hydride ion involved in the formation of the intermediate *I* was compared between the QM/MM model incorporating the effect of protein environment and the heme-thiolate active center model only dealing with the active center. Computed hydride affinities with both the QM/MM and active center models were similar to each other, implying that the protein environment of P450nor would provide an electrostatically weak effect on the active center. Previous studies showed that one of the structural characteristics of P450nor compared to other P450s is lack of a molecular dipole by the charge separation [31]. The charge separation observed in other P450s, positive proximal and negative distal, is suggested to be involved with proton/electron flow in their catalytic reactions [32]. These suggest a small effect of protein environment on the catalytic reaction in N_2_O generation process in P450nor, and it is possible that the reactions can be catalyzed by heme bound to Cys residue alone once the reaction elements are in place. Based on our database analysis and computation of hydride affinity and the previous studies [31,32], we can expect that only the heme-thiolate active center is able to form N_2_O from the intermediate *I* and that the reaction pathway obtained from the active center model provides a useful basis for understanding the details of the reaction mechanism, compared to future QM/MM calculations. In addition, a large number of possible pathways should also be examined in the search of the minimum energy pathway of the reaction. Here, we explored the reaction pathway for the formation of N_2_O from the Fe^3+^-NHO^•−^ type intermediate *I* and the second NO at the DFT level of theory, B3LYP, using the heme-thiolate active center model. Our computation demonstrated that the minimum energy pathway indicates that the formation process of N_2_O from intermediate *I* occurs through the steps of N–N bond formation followed by barrierless rearrangement, water-mediated proton transfer, rearrangement of Fe-ONNHO species, and dissociation of an N_2_O molecule.

Understanding the relationship between structures and function of proteins is a crucial scientific issue. It is also involved in the prediction of heme protein function since heme proteins serve diverse biological functions. We have been interested in the origin of the multifunctional nature of heme. Our previous studies have led to the working hypothesis that heme can catalyze a variety of reactions, controlled by proteins [33,34,35]. Our calculations using the active center model provided an energetically feasible reaction pathway. It indicates that that N_2_O can be produced even in a model consisting solely of the active center, reinforcing our working hypothesis, and it can potentially provide insights into the prediction of protein function, such as catalytic reactions.

## 2. Results and Discussion

### 2.1. Assessment of the Validity of Computational Model for the N_2_O Gereration from the Intermediate I of P450nor

#### 2.1.1. Comparison of Heme Distortions Using the Heme Database, PyDISH

**Figure 2:**
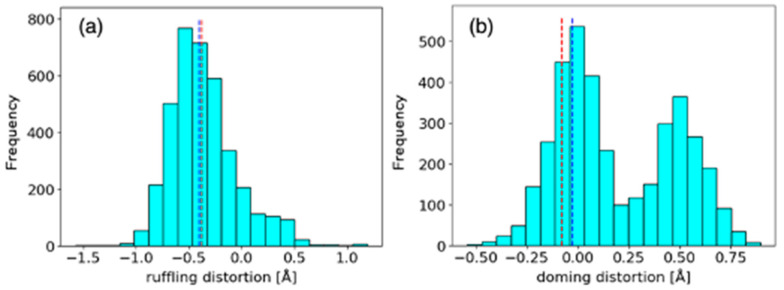
Distributions of (**a**) ruffling and (**b**) doming distortion on porphyrin scaffold of heme of oxidoreductases. The corresponding values for XFEL structures of P450nor in NO-bound (PDB ID: 5y5h) and the intermediate (PDB ID: 7dvo) states are shown in blue and red dashed lines, respectively.

To evaluate the validity of the active center model for the formation of N_2_O from intermediate *I*, we first evaluate the contribution of steric effect of protein environment to the reaction by performing a comparison of heme distortions using the heme database, PyDISH (https://pydish.bio.info.hiroshima-cu.ac.jp/, accessed on 18 October 2023). Heme distortion is strongly influenced by the protein environment and is related to heme functions such as redox potential and substrate binding [33,36]. Since P450nor only catalyzes NO reduction, unlike other P450 family members that catalyze oxygen addition reactions, the comparison also provides a clue to the origin of the specificity of P450nor function. Any heme distortion can be expressed by a linear combination of the vibrational modes of heme porphyrins [37]. In this study, we focused on the ruffling mode, which affects redox reactions [33], and the doming mode, which is associated with substrate binding [36]. Histograms of the results obtained from PyDISH are shown in Figure 2. Ruffling distortion (Figure 2a) showed a unimodal distribution, while doming distortion (Figure 2b) showed bimodal distributions, one for penta-coordinated heme and the other for hexa-coordinated heme. Hexa-coordinated hemes containing P450nor heme exhibited smaller doming distortion distribution in Figure 2b. In the NO-bound and intermediate states of P450nor, the ruffling and doming distortions of heme were evaluated to be −0.374 and −0.401, and −0.026 and −0.079, respectively. As shown in Figure 2, the ruffling and doming distortions of heme porphyrin in P450nor are located near the peak of the distributions, meaning that these structural parameters are similar in heme oxidoreductases and do not strongly suggest the differences in function between P450nor and other P450s.

**Figure 3:**
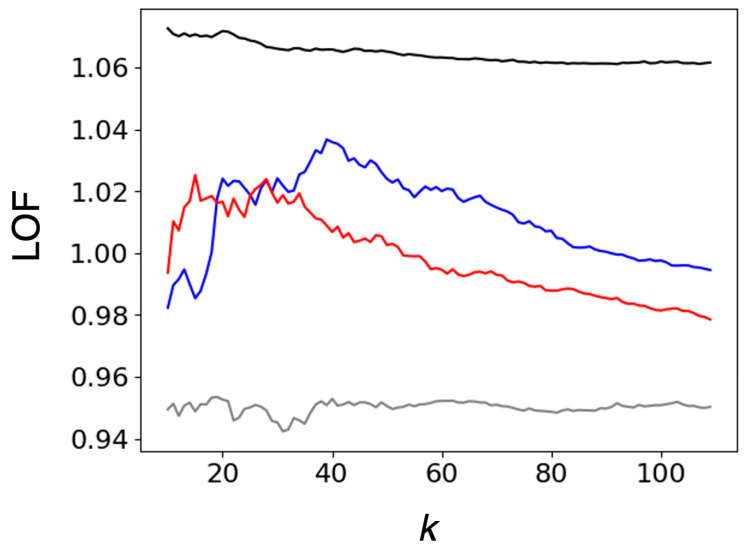
LOF plots versus parameter *k*. The blue and red lines represent the LOFs of XFEL structures of P450nor in NO-bound (PDB ID: 5y5h) and the intermediate (PDB ID: 7dvo) states, respectively. The median and minimum values of LOFs are also shown by black and gray lines, respectively.

To discuss the characteristics of heme porphyrin distortion of P450nor more quantitatively, the LOFs of P450nor were calculated. Since the LOFs are strongly dependent on parameter *k* that determines the size of cluster to evaluate the local density around the target data point, we plotted LOFs versus *k* rather than using a fixed *k* value. Figure 3 shows the median (black line) and minimum (gray line) LOF values for oxidoreductases (whole dataset). As illustrated in Figure 3, the LOF of P450nor was around 1 and smaller than the median for oxidoreductase, suggesting that the distortion parameter was inlier. The maximum LOF values for P450nor in the NO-bound and intermediate states were 1.037 and 1.025, corresponding to the 21 and 16 percentiles, respectively. P450nor has a common heme distortion as oxidoreductase both in the NO-bound and intermediate states. Heme distortions caused by the steric protein environment of P450nor do not significantly contribute to the unique reactivity of P450nor, implying that the reactivity should be attributed to other features.

#### 2.1.2. Hydride Affinity of the NO-Bound State with or without the Protein Environment of P450nor 

**Figure 4:**
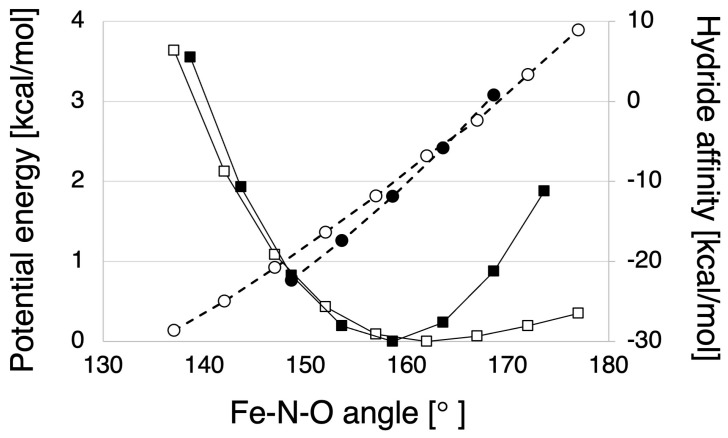
Hydride affinity plot versus Fe-N-O bonding angle (circles and dashed lines). The corresponding potential energy curves are also shown by squares and solid lines [23]. The computed hydride affinities and potential energies were listed in Table S1 in the Supplementary Materials. The filled and open markers represent the results for with and without protein condition, respectively.

The above analysis showed that the steric character of P450nor does not strongly affect unique reactivity. Next, to clarify the electrostatic effects of protein environments of P450nor, hydride affinities were compared between the NO-bound heme-thiolate complexes with the P450nor protein environment (QM/MM model) and without it (active center model). In Figure 4, the hydride affinities and potential energies [23] are plotted versus the Fe-N-O bond angle for the QM/MM and active center models. As described in the previous study [23], the NO molecule on the heme of P450nor has steric repulsion with the protein, mainly the carbonyl group of Ala239, against the angular change of the Fe-N-O angle, preventing the Fe-N-O moiety from taking an angle near 180 degrees, where hydride transfer is unfavorable. On the other hand, the hydride affinity of the NO-bound heme complex was found to be almost unchanged with or without protein environment, suggesting that the electrostatic field of P450nor has little effect on the active site.

These findings suggest that the protein environment of P450nor would not significantly function to promote the N_2_O formation reaction process, implying that the reaction would be able to proceed only with heme-specific activity. Furthermore, there are many possible pathways that need to be examined in order to find the minimum energy reaction pathway. Here, we employed the active center model as the first step to investigate the reaction mechanism of the N_2_O formation process from the intermediate *I*. Comparison of the reaction pathways obtained from the active center model with those from future QM/MM calculations will provide a detailed understanding of the catalytic reaction mechanism of P450nor, including the role of proteins.

### 2.2. Computed Reaction Mechanism of the N_2_O Formation from the Intermediate I of P450nor

**Figure 5:**
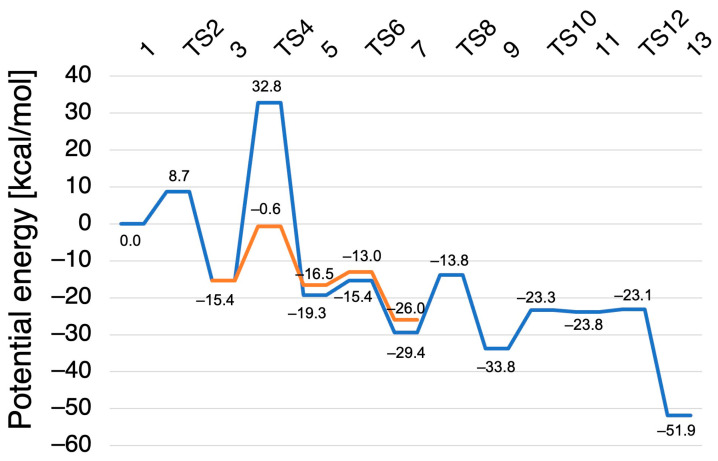
Potential energy profile and relative potential energies for the N_2_O formation reaction catalyzed by P450nor. The minimum energy pathway is shown in blue, and the water-mediated pathway is shown in orange.

**Figure 6:**
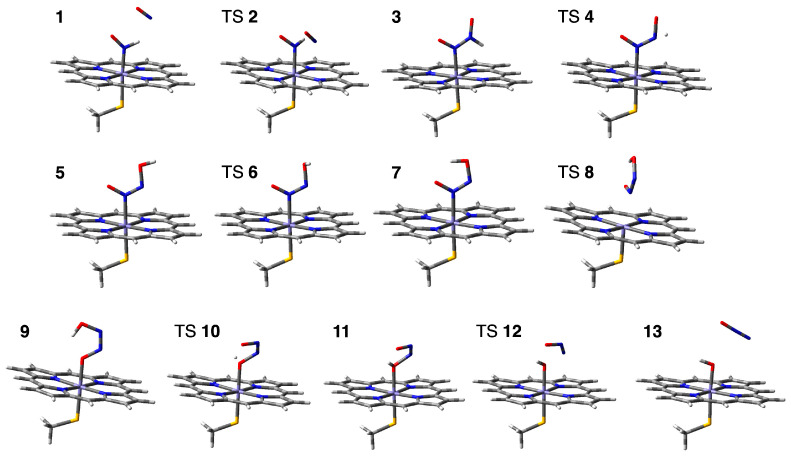
Optimized molecular structures of the stationary points in the reaction pathway from complex **1**, Fe^3+^-NHO^•−^ + NO^•^, to **13**, Fe^3+^-OH^−^ + N_2_O. The coordinates of the optimized structures are shown in Table S2 in the Supplementary Materials.

**Figure 7:**
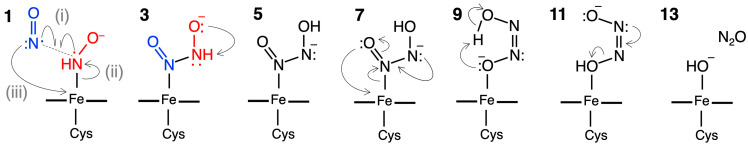
The schematic illustration of the reaction pathway from complex **1**, Fe^3+^-NHO^•−^ + NO^•^, to **13**, Fe^3+^-OH^−^ + N_2_O. NHO^•−^ and second NO molecule of complexes **1** and **3** are shown in red and blue, respectively. Note that the first step consists of three events: (i) N–N bond formation, (ii) breaking of the Fe–N coordination bond, and (iii) Fe–N coordination of the second NO part of ONHNO^−^.

The reaction of N_2_O formation from intermediate *I* was explored at the B3LYP level using the active center model. Figure 5 shows the minimum energy pathway. Figure 6 shows the corresponding structures of the stationary points in the pathway. Figure 7 shows the corresponding reaction scheme. The reaction pathway was found to consist of four steps: (1) N–N bond formation followed by barrierless rearrangement from complex **1** to **3**, (2) water-mediated proton transfer from complex **3** to **7**, (3) rearrangement of Fe-ONNHO species from complex **7** to **9**, and (4) N–O bond dissociation from complex **9** to **13**. We have also found metastable pathways with relatively higher barriers, which will be discussed later.

#### 2.2.1. N–N Bond Formation Followed by Barrierless Rearrangement Step

In the N-N bond formation followed by the barrierless rearrangement step, an N–N covalent bond was formed between NHO^•−^ group of intermediate *I* and the second NO molecule to yield a deprotonated hyponitrous acid, ONHNO^−^, followed by a barrierless rearrangement, where the NHO part (red in Figure 7) of ONHNO^−^ was dissociated from the heme Fe ion and the NO part (blue in Figure 7) was coordinated to it. 

When the second NO molecule approached to the intermediate, they formed complex **1**, where the NO molecule was fixed near the heme by the hydrogen bond with N…N distance of 2.80 Å. Next, NO directly attacked the N atom of the NHO^•−^ group of intermediate *I* through transition state (TS) **2** with a barrier of 8.5 kcal/mol, forming complex **3** consisting of an ONHNO^−^ species bound on heme. It should be noted that the Fe–N bond of NHO^•−^ group was broken after TS **2** by the N–N bond formation, and barrierless took place with a new Fe–N bond for the second NO moiety of the hyponitrous acid. As shown in Figure 7, during the elementary reaction from complex **1** to **3**, three events sequentially occur: (i) N–N bond formation between the second NO attack and the NHO group, (ii) the breaking of the Fe–N coordination bond between the NHO part of ONHNO^−^ and the heme Fe ion, and (iii) Fe–N coordination of the second NO part of ONHNO^−^ to the heme Fe ion. TS **2** was characterized by an imaginary frequency of 291*i* cm^−1^ (Figure S1) and was associated with the N–N stretching mode. After TS **2**, where the N–N distance was 2.04 Å, the N–N covalent bond was formed with distance of 1.31 Å in complex **3.** Due to the covalent bond formation, complex **3** was stabilized by 15.4 kcal/mol from the initial complex **1**. 

**Figure 8:**
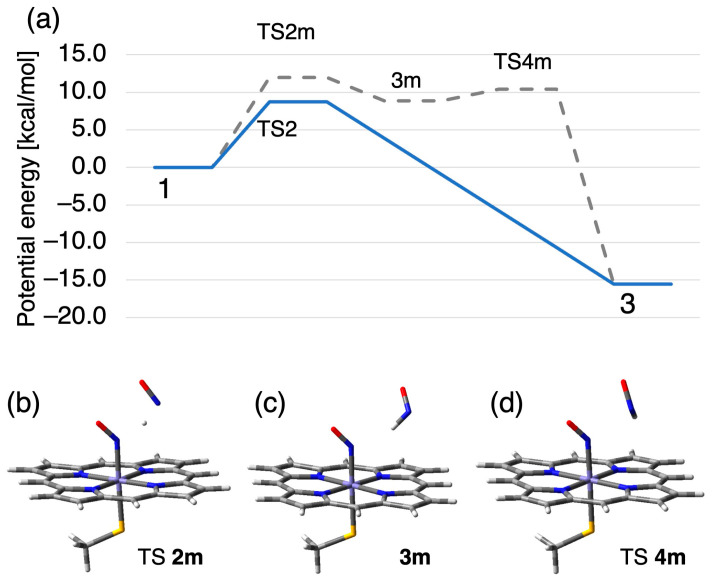
(**a**) Detailed potential energy profile of the metastable pathway (gray dashed line) for N–N bond formation from complex **1** to **3**, and (**b**–**d**) the corresponding stationary molecular structures.

We also found a metastable pathway for the N–N bond formation where the hydrogen atom of NHO^•−^ group transiently transfer to NO molecule, as shown in Figure 8. In this metastable pathway, the second NO molecule directly pulled the H atom from the NHO^•−^ group of intermediate *I* through transition state (TS) **2m** with a barrier of 12.0 kcal/mol, forming complex **3m** consisting of an Fe^3+^-NO^−^ species and HNO. Complex **3m** is 8.9 kcal/mol above complex **1**. In complex **3m**, the HNO molecule is bound to NO molecule on heme with weak hydrogen bond at a N–N distance of 3.11 Å. Subsequently, rebound of the NHO group to the Fe^3+^-NO^–^ species formed an N–N bond via TS **4m**, providing Fe^3+^-ONNHO^−^ (hyponitrous acid) species **3** to join up with the main pathway. TS **4m** has an imaginary frequency of 51.6*i* cm^−1^ associated with N–N bond formation by nucleophilic attack and is 1.5 kcal/mol above complex **3m**.

The bottleneck reaction barriers for the main and the metastable pathways for N–N bond formation were 8.5 and 12.0 kcal/mol, respectively. While the former is preferable, both pathways are acceptable with respect to the experimental reaction rate constant of 1200 s^−1^, which corresponds to the barrier height of 12.6 kcal/mol [21]. However, since the latter transiently yields an HNO species disbound from heme that is too reactive and thus harmful for living cell, it could be expected that the latter pathway would be forbidden in the enzymatic environment. A possible inhibitor for the HNO generation in P450nor is the carbonyl group of Ala239 that is vicinal to the heme, whose role was assigned to the steric control of Fe–N–O bending angle in our previous QM/MM study [23]. It is therefore expected that the Ala239 would suppress the metastable pathway by the steric repulsion as it does the Fe–N–O bending.

#### 2.2.2. Water-Mediated Proton Transfer Step

**Figure 9:**
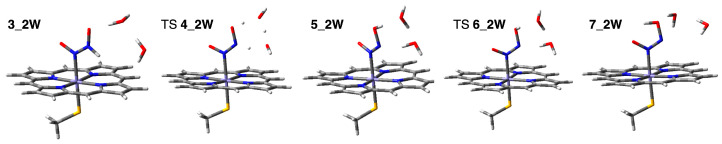
The optimized molecular structures of the stationary points in the two-water-mediated pathway from complex **3** to **7**.

In the next step, tautomerization of hyponitrous acid of species **3** occurred from ONHNO^−^ to HONNO^−^ via proton transfer. We first examined the intramolecular proton transfer without the aid of proton mediators to find an unrealistic barrier height of 48 kcal/mol, suggesting that the proton mediator is indispensable for this reaction. We then examined the proton transfer of hyponitrous acid by the extended system with two water molecules. The corresponding energy profile is shown in orange in Figure 5, and the stationary structures are shown in Figure 9. Note that hereafter the suffix of “**_2W**” is used for this additional-two-water models. The approach of two water molecules to species **3** led to a complex **3_2W**. In the complex **3_2W**, two hydrogen bonds are formed; one is between hyponitrous acid and water molecule with the O–O distance of 2.64 Å, and the other is between two water molecules with the O–O distance of 2.82 Å. It should be noted that the hydrogen bond between the NH moiety of the hyponitrous acid and the water molecule is not formed, presumably due to the steric repulsion between the heme and the water molecule. According to our calculations, this proton transfer formed a complex **5_2W** with an Fe^3+^-ONNOH^−^ species via TS **4_2W** with an energy barrier of 12.4 kcal/mol. The complex **5_2W** is 1.1 kcal/mol more stable than **3_2W**. The OH bond of complex **5_2W** was then flipped across TS **6_2W** with a barrier of 5.5 kcal/mol, resulting in the formation of an intramolecular hydrogen bond in the Fe^3+^-ONNOH^−^ species of the complex **7_2W**. TS **6_2W** was identified with an imaginary frequency of 210*i* cm^−1^ with respect to the concerted proton shuttle. The complex **7_2W** was 11.8 kcal/mol below complex **8** due to the formation of intramolecular hydrogen bond.

The potential energy profiles of the two pathways were compared to evaluate the contribution of the two waters for the reaction energy profile. It can be found in Figure 5 that while the two waters significantly stabilized the transition state of the proton transfer, they have a minor impact on the other states including OH bond rotation. Therefore, we concluded that the water molecules are indispensable only as mediators of the proton transfer reaction, and the following reaction pathway was analyzed without the water molecules for convenience.

#### 2.2.3. Rearrangement of the Fe-ONNHO Species

In the Fe^3+^-ONNOH^–^ species of complex **7**, the N atom of ONNOH^–^ is coordinated to Fe-porphyrin. In the next step, the ONNOH^−^ group was rearranged from the coordination at the N atom to the coordination at the O atom (species **9**) through TS **8**, which is above 15.6 kcal/mol. The rearrangement of species **7** to species **9** stabilized by 4.4 kcal/mol can be attributed to the bond switch from Fe–N to Fe–O. In TS **8**, the coordination bond lengths of Fe–N and Fe–O are 2.50 and 2.56 Å, respectively. It should be noted that the N–O bond to be cleaved in the next step was elongated by the rearrangement; the N–O bond lengths for species **7** and **9** were 1.29 and 1.34 Å, respectively.

#### 2.2.4. O–N Bond Dissociation Step

In the last step, the elongated N–O bond of ONNOH^–^ species was cleaved. Before the bond cleavage, proton transfer occurred within the ONNOH^−^ molecule, thereby generating N_2_O molecule by cleaving the O–N bond of ONNOH^−^. This reaction was proceeded by a stepwise mechanism in which the proton of the ONNOH^−^ moiety was transferred to the O atom coordinating the Fe ion and N_2_O dissociated. Species **11** was formed via TS **10** with a barrier of 10.5 kcal/mol. Finally, N_2_O is released through TS **12** with a barrier of only 0.3 kcal/mol and stabilizes 28.1 kcal/mol below species **13**. The overall reaction is 51.9 kcal/mol exothermic. The remaining OH^–^ group would take up a proton to form H_2_O and return to the resting state. 

**Figure 10:**
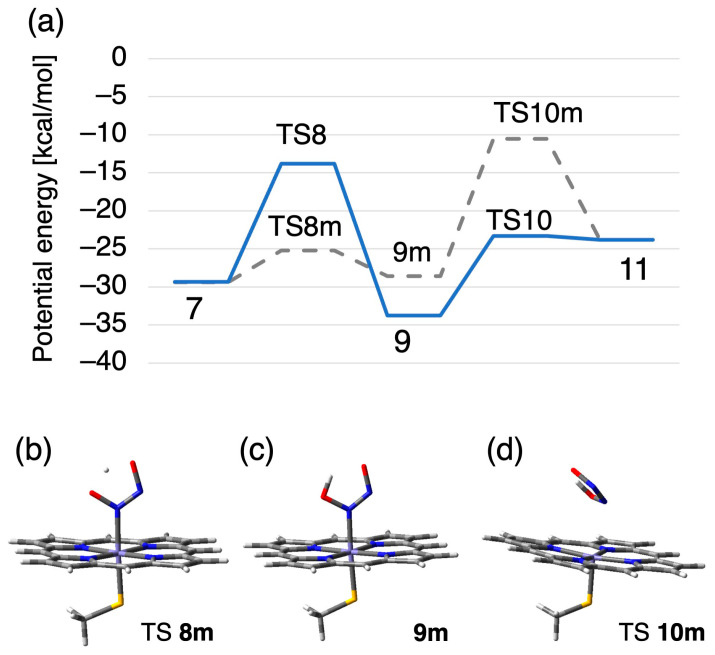
(**a**) Detailed potential energy profile of the metastable pathway (dashed line) for rearrangement and N_2_O dissociation from complex **7** to **11**, and (**b**–**d**) the corresponding stationary molecular structures.

We have also examined another pathway from species **7** to **11,** as shown in Figure 10. The difference between the metastable pathway shown in gray dashed lined and the above-mentioned main pathway is the order of the two elementary reactions. While the main reaction was achieved at first with the rearrangement of Fe–N bond with Fe–O bond and then the intramolecular proton transfer from Fe-ONNOH^−^ to Fe-OHNNO^−^, the proton transfer precedes in the metastable reaction via TS **8m** with a barrier height of 4.2 kcal/mol to form species **9m** that is slightly unstable than **7** with 0.8 kcal/mol. The bottleneck of the metastable pathway is again the bond rearrangement from Fe–N to Fe–O whose barrier height is 18.0 kcal/mol, which is higher than that of TS **8**.

## 3. Materials and Methods

### 3.1. Comparison of the Active Site Structure of P450nor to Those of the CYP Family with Heme Protein Database, PyDISH

In the CYP families, P450nor differs from the others in its ability to reduce NO to N_2_O. To evaluate the contribution of the steric effect of protein environment in P450nor, we first compared the heme distortions, directly and sterically caused by the protein environment, between P450nor and other oxidoreductases using PyDISH, a database of heme structures in heme proteins registered in the Protein Data Bank (PDB). As of 18 October 2023, 17,330 heme structures were deposited from 6517 PDB entries. From the registered structural data in PyDISH, a heme protein structural database that we have recently developed and released at https://pydish.bio.info.hiroshima-cu.ac.jp/ (accessed on 18 October 2023), 3739 entries with oxidoreductase activity as protein function, cysteine as axial ligand, and resolution lower than 3.0 Å were compared to the X-ray free electron laser (XFEL) structure of P450nor without radiation damage (PDB ID 5y5h and 7dvo) with respect to heme porphyrin distortions (ruffling and doming distortions). To quantitate the structural characteristics of the porphyrin distortion of P450nor, the local outlier factor (LOF) [38] for each data point *p* was calculated using scikit-learn. LOFs are evaluated via the local reachability density (lrd) and reachability distance (rd) of *p* from *q* according to the following equations:

(1) $$LOF_{k}\left(p\right)=\frac{1}{\left|N_{k}\right|}\sum_{i\in N_{k}}\frac{lrd_{k}(p_{i})}{lrd_{k}(p)},$$

(2) $${lrd}_{k}\left(p\right)={\left(\frac{1}{\left|N_{k}\right|}\sum_{i\in N_{k}}{rd}_{k}\left(p,p_{i}\right)\;\right)}^{-1},$$

(3) $${rd}_{k}\left(p,q\right)=max\left(distance\left(p,q\right),distance\left(q,q_{k}\right)\right),$$

where $N_{k}$ represents the set of *k* nearest neighbors, $\left|\cdot \right|$ represents the size of the set $N_{k}$, and $p_{i}$ and $q_{i}$ are the *i*-th nearest neighbor points for *p* and *q*, respectively. Note that LOF values depend on the parameter *k* that define the cluster size to evaluate the local density.

### 3.2. QM/MM Calculation for Hydride Affinity Evaluation

We applied the QM/MM model that have been constructed in our previous study [23], where the QM region including A heme, NO, Cys352, and main chains of Ile353, Ala354, and Glu355 were treated with B3LYP/def2svp level of calculation and the remaining portion was treated with Amber ff96 force field. The hydride affinity was evaluated by the energy change due to the hydride transfer from NADH to the NO-bound state of P450nor, Fe^3+^-NO, namely the energy change from NADH + Fe^3+^-NO to NAD^+^ + Fe^3+^-NHO^•−^. It should be noted that the atomic coordinates, except for hydride, for each bending angle were same as those of our previous study [23], while the position of the hydride was optimized in the present study. The Conductor-like Polarizable Continuum Model (CPCM) [39] was applied to take account of the polarization effect of the aqueous solution, with the scaling factor of 1.8 for cavity radius to avoid the charge penetration of CPCM into the protein core region.

### 3.3. Quantum Chemical Exploration of a Reaction Path of the N_2_O Generation

In the exploration of the reaction pathway from intermediate *I* to N_2_O formation, we first constructed a model for intermediate *I*. The active center of P450nor consists of a heme *b* with Cys coordinated as the axial ligand. We constructed a model of the active center in which heme *b* is replaced by Fe^3+^-porphyrin and axially ligated Cys by CH_3_S^−^. Since our time-resolved IR spectroscopy, X-ray free electron laser crystallography, and computational studies have revealed that the intermediate I is an Fe^3+^-NHO^•−^ species [28], we built a model of intermediate *I* in which NHO^•−^ is bound to the model of the active center. In the present study, we did not consider the effects of proteins surrounding the active center, since we know that they do not significantly affect its molecular structure from previous studies on intermediates. The charge and spin multiplicity of the system was set as −1 and 2 (doublet), respectively. We confirmed that the doublet state of complex **1** was more stable than the quartet state by 9.8 kcal/mol. The reaction of N_2_O production with the intermediate *I* model was studied using the B3LYP exchange–correlation functional [40]. As basis sets, the Pople 6-311++G (2d, 2p) [41] was used for Fe, N, S, O atoms and hydride atom in NHO^•−^, and the def2-svp basis sets [42] for the others. All stationary points and transition states in the reactions were characterized by frequency calculations at the same computational level, while the marginal imaginary frequency less than 30*i* cm^–1^ was ignored, which was attributed to the rotation of the CH_3_S^−^ group. All calculations were performed using the Gaussian09 program suite [43]. Although the present level of calculation has not been tailored for the reaction energy but for the molecular geometry, vibrational frequency, and p*K*_a_ in the previous studies [23,28], we evaluated the barrier height for TS2 using the single point MP2 level of calculation with the same basis set to evaluate the robustness of the present results. The resulting value was 12.2 kcal/mol, which reasonably agreed with that of B3LYP level of calculation, 8.7 kcal/mol.

## 4. Conclusions

In the present study, we have found the minimum energy reaction pathway of the N_2_O generation from the intermediate *I* of P450nor, which catalyzes the conversion of NO to N_2_O with two-electron reduction, at the B3LYP level of theory, using an active site model. First of all, the active center model of P450nor was examined for validity to explore the reaction pathway with both database analysis and DFT calculations. In the first step of the validation, database analysis was performed to evaluate the contribution of the steric effect of protein environment. We compared the heme distortions related to the redox potential between P450nor and other oxidoreductases, including P450s, using PyDISH [30] and showed that the steric protein environment of P450nor would not be attributed to its characteristic catalytic ability in converting intermediate *I* to N_2_O. In the second step of the validation, we evaluate the electrostatic effect of protein environment on the reaction using DFT calculations. Similar hydride affinities calculated with both the QM/MM and active center models indicated that electrostatic effects of protein environment of P450nor weakly influence the reactivity of the active center. These results are consistent with the previously reported structural characteristics of P450nor [31] and lead us to expect that the reaction can proceed only with the active center. Next, we explored the reaction pathway in the N_2_O formation from the Fe^3+^-NHO^•−^ type intermediate *I* and the second NO at the DFT level of theory, B3LYP, using the active center model as the first step to understand the reaction mechanism of the formation of N_2_O from the intermediate *I*. We showed that the minimum energy pathway composed of N–N bond formation followed by barrierless rearrangement, water-mediated proton transfer, rearrangement of Fe-ONNHO species, and dissociation of an N_2_O molecule. It indicates that the heme-thiolate active center model can convert the intermediate *I* into N_2_O. Future QM/MM calculations that include the effect of the protein environment and comparison with the present results will provide more details on the catalytic reaction mechanism of P450nor and reveal how the protein regulates the catalytic reaction.

Structure–function relationships have been an important scientific issue for a long time, which is also related to the prediction of protein function. Heme proteins have diverse biological functions. We have performed research in computational chemistry as well as machine learning with the goal of understanding the origin of the multifunctional nature of heme [33,34,35]. From this viewpoint, our result that N_2_O can be formed even in a model consisting only of the active center enhances our working hypothesis derived from our previous studies that heme itself can catalyze a variety of reactions, controlled by proteins [33,34,35]. This study can potentially be applied to predict protein function, such as catalytic reactions. This is the first step toward understanding structure–function relationships in the active sites of heme proteins. 

## Data Availability

The atomic coordinates of P450nor were downloaded from PDBj (https://pdbj.org/, accessed on 18 October 2023). The statistical data of heme porphyrin distortion associated with the functions are available on the PyDISH website (https://pydish.bio.info.hiroshima-cu.ac.jp, accessed on 18 October 2023).

## Supplementary Materials

The following supporting information can be downloaded at https://www.mdpi.com/article/10.3390/ijms242417172/s1.
