# Supplementary material for: Computational Exploration of Minimum Energy Reaction Pathway of N2O Formation from Intermediate I of P450nor Using an Active Center Model
Source: Int J Mol Sci. 2023 Dec 6;24(24):17172. doi: 10.3390/ijms242417172 (PMC10743073; doi:10.3390/ijms242417172)
Supplement: Supplementary file 1 [file ijms-24-17172-s001.zip › ijms-2726505-supplementary.pdf]

Supplementary Materials

Computational Exploration of Minimum Energy Reaction Pathway of N_2O Formation from Intermediate *I* of P450nor Using an Active Center Model

Yusuke Kanematsu [1,2,*], Hiroko X. Kondo [2,3] and Yu Takano [2,*]

[1] Graduate School of Advanced Science and Engineering, Hiroshima University, 1-4-1 Kagamiyama, Higashi-Hiroshima 739-8527, Japan

[2] Graduate School of Information Sciences, Hiroshima City University, 3-4-1 Ozukahigashi Asaminamiku, Hiroshima 731-3194, Japan; h_kondo@mail.kitami-it.ac.jp

[3] Faculty of Engineering, Kitami Institute of Technology, 165 Koen-cho, Kitami 090-8507, Japan

* Correspondence: ykanem@hiroshima-u.ac.jp (Y.K.); ytakano@hiroshima-cu.ac.jp (Y.T.);

Table S1. Relative potential energy (ΔE, in kcal/mol) and hydride affinity (E_{hyd}, in kcal/mol) plotted for Fe-N-O bending angle.

Isolated			ONIOM		
A(Fe-N-O)	ΔE	E_{hyd}	A(Fe-N-O)	ΔE	E_{hyd}
132.0	5.7	-32.0	133.6	5.8	
137.0	3.6	-28.6	138.6	3.6	
142.0	2.1	-25.0	143.6	1.9	
147.0	1.1	-20.8	148.6	0.8	-22.4
152.0	0.4	-16.3	153.6	0.2	-17.4
157.0	0.1	-11.8	158.6	0.0	-11.9
162.0	0.0	-6.8	163.6	0.2	-5.8
167.0	0.1	-2.4	168.6	0.9	0.8
172.0	0.2	3.3	173.6	1.9	
177.0	0.4	8.9			

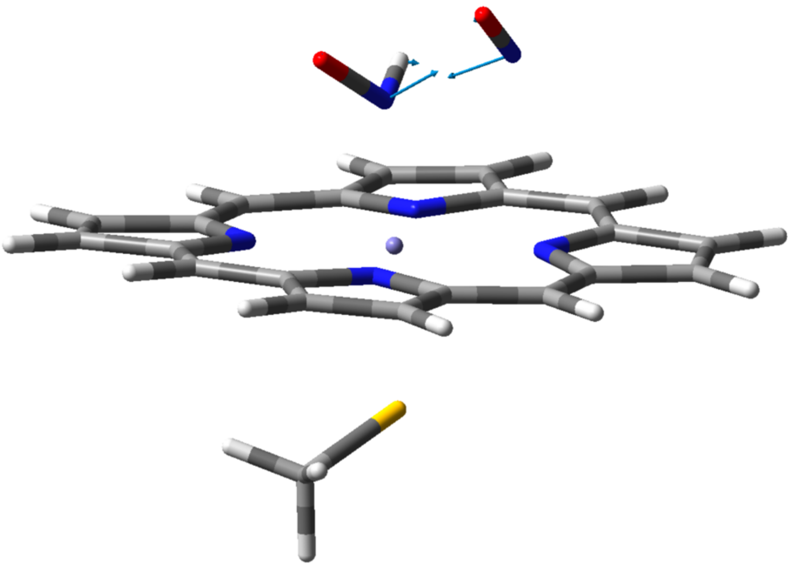

Figure S1. Imaginary frequency mode of TS **2** shown in blue arrows.

Table S2. Coordinates of the optimized geometries from the reactant (01) to the product (13).

01.xyz

Fe	0.036328	-0.000233	0.169274
C	-3.373309	-0.010844	0.719835
C	0.041954	-3.407326	0.204496
C	3.396485	0.010351	-0.523628
C	0.021544	3.406822	0.209495
N	-1.398235	-1.428439	0.402863
C	-2.735636	-1.246507	0.622539
C	-3.399136	-2.527239	0.758899
C	-2.434329	-3.481357	0.624690
C	-1.185863	-2.780171	0.401722
N	1.455138	-1.418611	-0.082207
C	1.260052	-2.770123	-0.028927
C	2.506250	-3.464562	-0.284715
C	3.448996	-2.504724	-0.506948
C	2.776417	-1.228248	-0.377786
N	1.446669	1.427070	-0.080065
C	2.769071	1.245039	-0.375819
C	3.434010	2.525718	-0.502965
C	2.485514	3.479562	-0.279378
C	1.243461	2.777300	-0.024765
N	-1.406828	1.419028	0.404878
C	-1.202520	2.772046	0.405777
C	-2.455146	3.465408	0.629718
C	-3.414230	2.505332	0.762620
C	-2.743098	1.228808	0.624325
H	-4.450571	-0.014229	0.898944
H	0.049569	-4.499510	0.222580
H	4.462119	0.013694	-0.762938
H	0.022599	4.499004	0.229157
H	-4.466101	-2.658530	0.937818
H	-2.539051	-4.565307	0.667406
H	2.622244	-4.548145	-0.297688
H	4.506606	-2.629839	-0.738240
H	4.490883	2.657517	-0.733919
H	2.595028	4.563838	-0.290700
H	-2.566362	4.548647	0.674010
H	-4.481944	2.629992	0.941810
N	-0.305091	0.000735	-1.692197
O	0.461173	0.001322	-2.674418
S	0.293781	-0.000093	2.493153
C	2.074863	-0.000370	2.887289
H	2.581548	0.891981	2.488103
H	2.184970	0.000783	3.984179
H	2.580918	-0.893939	2.490030
H	-1.320108	0.001308	-1.975582
N	-2.387730	0.001911	-3.561228
O	-1.565789	0.002295	-4.381796

02ts.xyz

Fe	-0.030167	0.097094	-0.099474
C	2.450116	2.490908	-0.161856
C	2.318016	-2.336206	-0.524836
C	-2.469182	-2.285775	0.206163
C	-2.381001	2.555046	0.197775
N	1.989150	0.085905	-0.308053
C	2.834583	1.161129	-0.313020

C	4.199379	0.724296	-0.525141
C	4.158309	-0.631996	-0.652056
C	2.770004	-1.019451	-0.505914
N	-0.071062	-1.915462	-0.149079
C	0.998047	-2.745092	-0.339444
C	0.572163	-4.129369	-0.294120
C	-0.771704	-4.116174	-0.064979
C	-1.162832	-2.724712	0.018159
N	-2.043651	0.124106	0.107951
C	-2.873229	-0.952823	0.234286
C	-4.243991	-0.512997	0.392590
C	-4.219441	0.850206	0.365863
C	-2.832168	1.235889	0.202407
N	0.033106	2.131097	0.028928
C	-1.051313	2.963601	0.124267
C	-0.617068	4.344682	0.152112
C	0.744936	4.328295	0.068920
C	1.137022	2.937417	-0.017639
H	3.237735	3.247041	-0.187449
H	3.065222	-3.118284	-0.675968
H	-3.242772	-3.047562	0.324290
H	-3.137474	3.338836	0.279299
H	5.063414	1.386758	-0.571973
H	4.981990	-1.325483	-0.819753
H	1.233853	-4.986888	-0.414379
H	-1.452969	-4.960201	0.039985
H	-5.100343	-1.176490	0.510694
H	-5.051073	1.548073	0.461420
H	-1.283361	5.203972	0.225915
H	1.436003	5.170972	0.056737
N	0.023127	0.101028	1.907359
O	-0.686195	-0.630161	2.653716
S	-0.231806	0.346538	-2.402884
C	-1.418046	-0.897402	-3.010219
H	-2.414997	-0.751866	-2.566031
H	-1.505368	-0.789550	-4.103701
H	-1.082596	-1.921534	-2.786255
H	0.158709	1.055778	2.278236
N	1.904019	-0.399067	2.506899
O	1.672302	-1.278927	3.221713

03.xyz

Fe	0.001579	0.000556	-0.131030
C	3.446495	0.083650	-0.419904
C	0.098664	-3.406426	-0.122046
C	-3.401248	-0.081383	0.263826
C	-0.064206	3.408015	-0.104704
N	1.492426	-1.386379	-0.222821
C	2.837165	-1.169493	-0.366506
C	3.541342	-2.430661	-0.454310
C	2.596402	-3.410264	-0.370325
C	1.318510	-2.744939	-0.230472
N	-1.387195	-1.455198	0.012479
C	-1.150826	-2.801064	-0.002424
C	-2.394606	-3.527775	0.150026
C	-3.380224	-2.593398	0.267671
C	-2.735215	-1.300402	0.183863
N	-1.455292	1.387608	0.014075
C	-2.794435	1.167893	0.185355
C	-3.500826	2.428138	0.273865

C	-2.561111	3.409075	0.161026
C	-1.283919	2.743054	0.006590
N	1.423909	1.457063	-0.213530
C	1.185793	2.805673	-0.212394
C	2.431086	3.531996	-0.344708
C	3.421402	2.599030	-0.433633
C	2.777648	1.305179	-0.356101
H	4.532031	0.110303	-0.533263
H	0.123377	-4.498168	-0.126823
H	-4.483579	-0.107507	0.405126
H	-0.091375	4.499705	-0.102774
H	4.620282	-2.532956	-0.566402
H	2.733208	-4.490868	-0.398422
H	-2.479707	-4.613850	0.172948
H	-4.450032	-2.745803	0.407052
H	-4.576697	2.528364	0.413902
H	-2.697868	4.489729	0.189289
H	2.516369	4.618061	-0.365007
H	4.494470	2.753066	-0.542905
N	1.215972	-0.003609	2.533667
O	1.442497	-0.003544	3.788660
S	-0.041049	-0.006487	-2.416676
C	-1.775778	0.004659	-2.970739
H	-2.303521	0.905917	-2.624723
H	-1.781156	-0.004976	-4.072541
H	-2.319782	-0.880762	-2.609375
H	1.990457	-0.007617	1.876445
N	0.051734	-0.002145	1.935450
O	-0.976782	-0.001208	2.650352

03_2W.xyz

Fe	0.487106	-0.037628	-0.280360
C	-2.101789	-2.068565	-1.338976
C	-1.416943	2.715592	-0.966308
C	2.979027	1.974727	0.937271
C	2.359073	-2.793870	0.405515
N	-1.409209	0.258388	-0.985787
C	-2.300065	-0.692293	-1.420259
C	-3.476026	-0.058314	-1.970692
C	-3.278190	1.290524	-1.869147
C	-1.982775	1.477026	-1.256117
N	0.734203	1.951600	-0.049140
C	-0.163935	2.926393	-0.394579
C	0.371794	4.231923	-0.073790
C	1.604371	4.026368	0.472400
C	1.820584	2.596472	0.478869
N	2.331346	-0.340505	0.466255
C	3.210183	0.603666	0.920545
C	4.420474	-0.028027	1.402665
C	4.248262	-1.369532	1.240603
C	2.931108	-1.551415	0.664828
N	0.167890	-2.037144	-0.401857
C	1.074506	-3.011464	-0.084006
C	0.510073	-4.320654	-0.335489
C	-0.748444	-4.117695	-0.817657
C	-0.947981	-2.684869	-0.861569
H	-2.906758	-2.713440	-1.696117
H	-2.009896	3.600606	-1.204927
H	3.768268	2.614178	1.337229
H	2.965539	-3.675942	0.620715

H	-4.332991	-0.589365	-2.383225
H	-3.939805	2.097956	-2.180827
H	-0.147844	5.174005	-0.245069
H	2.315311	4.763260	0.844465
H	5.274735	0.504242	1.819486
H	4.930215	-2.179781	1.496351
H	1.026905	-5.263285	-0.158513
H	-1.488651	-4.856299	-1.123336
N	-1.482123	-0.025591	1.939879
O	-2.069705	-0.004164	3.097896
S	1.199969	-0.027595	-2.425822
C	2.745666	0.927768	-2.533903
H	3.544858	0.476041	-1.928225
H	3.061845	0.934525	-3.589086
H	2.596485	1.967076	-2.205366
N	-0.203311	-0.085176	1.728608
O	0.556602	-0.136303	2.715674
H	-2.019600	0.034065	1.080967
O	-5.967339	0.508279	0.515712
H	-5.545237	0.267010	1.364479
H	-5.232575	0.576996	-0.101214
O	-4.688219	-0.295295	2.902523
H	-3.700682	-0.145489	2.985920
H	-4.800474	-1.240206	3.027455

04ts.xyz

Fe	0.001989	0.018785	-0.154133
C	3.428374	0.219026	-0.442779
C	0.204761	-3.387399	-0.192744
C	-3.393292	-0.185213	0.316517
C	-0.185545	3.421105	-0.087528
N	1.526451	-1.318754	-0.272299
C	2.862277	-1.051947	-0.401537
C	3.611304	-2.285872	-0.502720
C	2.701416	-3.300124	-0.439471
C	1.400265	-2.681236	-0.298280
N	-1.338603	-1.487106	0.007992
C	-1.061211	-2.825567	-0.042779
C	-2.278318	-3.593773	0.114200
C	-3.290625	-2.693628	0.270708
C	-2.688885	-1.379607	0.204187
N	-1.501532	1.352259	0.049356
C	-2.829805	1.084780	0.240774
C	-3.577566	2.318727	0.349102
C	-2.674813	3.332992	0.223989
C	-1.378538	2.714165	0.043205
N	1.361949	1.521722	-0.238636
C	1.081658	2.859937	-0.217563
C	2.303725	3.628611	-0.335533
C	3.322483	2.728253	-0.429365
C	2.718608	1.413896	-0.369264
H	4.513109	0.283751	-0.549274
H	0.266080	-4.477440	-0.217924
H	-4.471717	-0.249196	0.474670
H	-0.248979	4.511232	-0.075481
H	4.694003	-2.348011	-0.608645
H	2.876162	-4.374894	-0.481038
H	-2.329328	-4.682263	0.113428
H	-4.352556	-2.882996	0.424579
H	-4.653704	2.380446	0.508324

H	-2.849345	4.408026	0.259237
H	2.354131	4.717070	-0.338738
H	4.391267	2.916272	-0.527094
N	1.326432	0.023106	2.456752
O	1.443237	-0.227458	3.868911
S	-0.094860	0.053980	-2.425498
C	-1.831173	-0.122429	-2.944954
H	-2.453545	0.695749	-2.553226
H	-1.859842	-0.090631	-4.045944
H	-2.256389	-1.080056	-2.609559
H	2.010392	-0.788206	2.835673
N	0.134549	-0.020090	1.935686
O	-0.868115	-0.032178	2.666775

04ts_2W.xyz

Fe	-0.380899	0.064718	-0.342188
C	2.273634	1.922225	-1.506902
C	1.262891	-2.802073	-1.182486
C	-2.984059	-1.786355	0.917220
C	-1.988133	2.934958	0.554763
N	1.426538	-0.351121	-1.161009
C	2.351840	0.539635	-1.634867
C	3.441852	-0.161759	-2.273404
C	3.160744	-1.493182	-2.173583
C	1.895296	-1.598724	-1.479779
N	-0.778461	-1.905805	-0.149297
C	0.025762	-2.936824	-0.557880
C	-0.598733	-4.205071	-0.254482
C	-1.793082	-3.921437	0.341109
C	-1.895014	-2.480861	0.398216
N	-2.160712	0.486953	0.511906
C	-3.099764	-0.402022	0.960893
C	-4.233824	0.303863	1.516769
C	-3.955378	1.633268	1.407343
C	-2.650780	1.733066	0.786451
N	0.088230	2.034474	-0.398943
C	-0.716058	3.065906	0.005681
C	-0.062967	4.333877	-0.237830
C	1.140004	4.050308	-0.813232
C	1.218290	2.610217	-0.918889
H	3.104704	2.511129	-1.898131
H	1.778454	-3.720499	-1.470197
H	-3.809879	-2.374871	1.321554
H	-2.507916	3.853682	0.834019
H	4.308560	0.319975	-2.723788
H	3.743255	-2.340512	-2.533851
H	-0.161635	-5.178902	-0.472855
H	-2.547289	-4.612283	0.716472
H	-5.114024	-0.175533	1.943913
H	-4.556966	2.484083	1.725696
H	-0.488733	5.306884	0.005430
H	1.916605	4.738712	-1.144484
N	1.642189	-0.137436	2.025082
O	1.961991	-0.314517	3.341503
S	-1.203951	0.182743	-2.431116
C	-2.856532	-0.579108	-2.465921
H	-3.571269	-0.027600	-1.838039
H	-3.207608	-0.553888	-3.509926
H	-2.823864	-1.626134	-2.130952
N	0.398252	-0.070821	1.718458

O -0.435193 -0.166688 2.636138
H 2.514427 -0.040711 1.354042
O 3.966784 0.083536 0.998267
H 4.379069 -0.227758 2.119376
H 4.244600 -0.560929 0.344026
O 4.519605 -0.501540 3.266550
H 2.990169 -0.390850 3.366000
H 4.942239 0.249981 3.688250

05.xyz
Fe 0.011497 -0.000066 -0.152263
C 3.442021 0.069196 -0.427505
C 0.085952 -3.409627 -0.152261
C -3.391625 -0.068455 0.294538
C -0.050256 3.410231 -0.136428
N 1.483126 -1.393822 -0.258615
C 2.827507 -1.178673 -0.378035
C 3.531917 -2.440832 -0.458987
C 2.585058 -3.419326 -0.388986
C 1.306795 -2.749526 -0.264419
N -1.385742 -1.451321 0.017757
C -1.158472 -2.799179 -0.013486
C -2.404462 -3.519072 0.147153
C -3.383069 -2.579266 0.284471
C -2.731389 -1.289869 0.203669
N -1.442745 1.393986 0.020349
C -2.780791 1.178299 0.207041
C -3.483499 2.440294 0.293970
C -2.543326 3.418968 0.160853
C -1.269649 2.749837 -0.003451
N 1.426177 1.452093 -0.249050
C 1.196034 2.799709 -0.248891
C 2.446705 3.520292 -0.369812
C 3.431754 2.580604 -0.444346
C 2.778285 1.291016 -0.369772
H 4.529151 0.091312 -0.528902
H 0.106756 -4.501532 -0.162588
H -4.472807 -0.090366 0.445525
H -0.072932 4.502134 -0.141286
H 4.612458 -2.543917 -0.556196
H 2.719283 -4.500514 -0.414724
H -2.496397 -4.604899 0.160601
H -4.452423 -2.726158 0.433889
H -4.557706 2.543575 0.445101
H -2.678311 4.500196 0.180062
H 2.537818 4.606075 -0.390512
H 4.507302 2.727145 -0.541097
N 1.293969 0.002714 2.416164
O 1.261158 0.001298 3.860085
S -0.073895 -0.006796 -2.435899
C -1.818586 0.012765 -2.958242
H -2.335350 0.916673 -2.602804
H -1.844231 0.002811 -4.059907
H -2.360394 -0.869603 -2.586397
H 2.207030 0.005386 4.033564
N 0.122871 -0.002692 1.946042
O -0.917517 -0.008655 2.653402

05_2W.xyz
Fe -0.413637 0.078083 -0.306101

C	1.442251	2.900564	-0.987527
C	2.054047	-1.862993	-1.633014
C	-2.149873	-2.739958	0.608343
C	-2.930584	2.025153	0.896118
N	1.403192	0.452695	-1.135119
C	1.978267	1.672168	-1.368479
C	3.225062	1.507904	-2.081989
C	3.386759	0.164114	-2.280106
C	2.240393	-0.483437	-1.679392
N	-0.106258	-1.911212	-0.464193
C	0.974799	-2.517184	-1.046524
C	0.846445	-3.956212	-0.958931
C	-0.326686	-4.205602	-0.309946
C	-0.916696	-2.918076	-0.013943
N	-2.214471	-0.293138	0.528372
C	-2.754932	-1.508727	0.840583
C	-4.054785	-1.337266	1.457033
C	-4.281320	0.004675	1.513695
C	-3.113833	0.647115	0.943845
N	-0.666824	2.071767	-0.052192
C	-1.784307	2.677340	0.449549
C	-1.612587	4.115392	0.456047
C	-0.373073	4.363511	-0.053720
C	0.203820	3.076132	-0.378534
H	2.025263	3.795641	-1.212780
H	2.831202	-2.486057	-2.080345
H	-2.691710	-3.635680	0.918419
H	-3.743800	2.649443	1.271997
H	3.874847	2.324721	-2.394467
H	4.196264	-0.355027	-2.792755
H	1.576162	-4.668406	-1.342973
H	-0.769414	-5.166179	-0.048311
H	-4.690954	-2.151862	1.801832
H	-5.143862	0.533138	1.918672
H	-2.356564	4.827002	0.813001
H	0.120765	5.322209	-0.209321
N	1.596557	-0.332519	1.825866
O	1.915519	-0.477931	3.208075
S	-1.307925	0.251996	-2.390001
C	-2.475379	-1.122802	-2.643682
H	-3.301348	-1.090799	-1.918027
H	-2.893109	-1.027720	-3.658713
H	-1.971304	-2.097407	-2.562071
N	0.381352	-0.000556	1.668407
O	-0.396081	0.189382	2.634287
H	3.165667	0.316917	1.128994
O	4.116332	0.561634	1.017999
H	4.796907	-0.428391	2.320434
H	4.265698	0.536295	0.067401
O	4.900714	-0.916496	3.166246
H	2.829354	-0.802916	3.168609
H	5.047830	-0.229831	3.821612

06ts.xyz

Fe	0.012976	0.005951	-0.156468
C	3.441823	-0.047945	-0.426342
C	-0.034775	-3.406670	-0.150293
C	-3.391009	0.056550	0.295100
C	0.071213	3.414994	-0.144439
N	1.432180	-1.440730	-0.254822

C	2.783836	-1.272633	-0.372959
C	3.443550	-2.558468	-0.450989
C	2.463014	-3.503437	-0.382048
C	1.208639	-2.789531	-0.260127
N	-1.435183	-1.396536	0.017310
C	-1.257025	-2.752005	-0.013627
C	-2.527859	-3.426779	0.147537
C	-3.472079	-2.452247	0.285057
C	-2.774741	-1.187226	0.203919
N	-1.390899	1.449459	0.023342
C	-2.736121	1.281219	0.207706
C	-3.393674	2.567298	0.291302
C	-2.419368	3.512051	0.156518
C	-1.170156	2.798663	-0.005664
N	1.476078	1.405407	-0.258496
C	1.294456	2.760289	-0.259762
C	2.570229	3.435870	-0.380428
C	3.520905	2.461803	-0.451312
C	2.821555	1.196503	-0.375434
H	4.529313	-0.064655	-0.523275
H	-0.052564	-4.498662	-0.161003
H	-4.472479	0.073388	0.444744
H	0.087402	4.506949	-0.151278
H	4.519992	-2.699062	-0.545925
H	2.559577	-4.588682	-0.407466
H	-2.658909	-4.508613	0.160696
H	-4.546009	-2.560650	0.434697
H	-4.463757	2.708928	0.440566
H	-2.516391	4.597371	0.172424
H	2.700015	4.517650	-0.400564
H	4.601357	2.569247	-0.543305
N	1.314865	-0.021794	2.418633
O	1.316070	0.026701	3.877508
S	-0.077199	0.027060	-2.434982
C	-1.819881	-0.058218	-2.957549
H	-2.397801	0.796398	-2.575456
H	-1.845174	-0.035452	-4.059004
H	-2.298682	-0.987263	-2.613973
H	1.480842	-0.890476	4.122444
N	0.131832	0.003419	1.956041
O	-0.911848	0.010902	2.645308

06ts_2W.xyz

Fe	-0.396925	0.067146	-0.324758
C	2.041629	2.238138	-1.416562
C	1.520952	-2.578307	-1.307315
C	-2.801897	-2.095798	0.824677
C	-2.265848	2.713493	0.740591
N	1.433233	-0.125890	-1.183948
C	2.264894	0.876090	-1.603275
C	3.433632	0.318428	-2.247659
C	3.291374	-1.040789	-2.203471
C	2.031888	-1.303548	-1.541447
N	-0.605040	-1.941196	-0.255189
C	0.298131	-2.865226	-0.707179
C	-0.198430	-4.204481	-0.472235
C	-1.415101	-4.069729	0.128486
C	-1.656283	-2.649281	0.260259
N	-2.199208	0.266112	0.567633
C	-3.044756	-0.733995	0.963265

C	-4.234964	-0.174157	1.567989
C	-4.084463	1.179922	1.540366
C	-2.802685	1.441255	0.917571
N	-0.136554	2.077149	-0.302275
C	-1.027162	2.999405	0.172745
C	-0.507279	4.338723	-0.010761
C	0.705852	4.206201	-0.617101
C	0.923310	2.786806	-0.798873
H	2.804720	2.927574	-1.782472
H	2.129976	-3.425957	-1.627997
H	-3.565103	-2.784483	1.192316
H	-2.867105	3.560176	1.078150
H	4.246176	0.904203	-2.676222
H	3.961232	-1.807365	-2.592082
H	0.333468	-5.118250	-0.735630
H	-2.097979	-4.848952	0.465605
H	-5.062299	-0.759843	1.967504
H	-4.761266	1.948558	1.912466
H	-1.021486	5.250350	0.292162
H	1.405341	4.984441	-0.920528
N	1.574504	0.426756	1.863401
O	1.941677	0.359100	3.253619
S	-1.274707	0.230584	-2.414007
C	-2.785724	-0.780742	-2.506559
H	-3.553649	-0.427509	-1.802584
H	-3.181914	-0.699797	-3.531442
H	-2.573816	-1.839014	-2.294002
N	0.421965	-0.081397	1.643477
O	-0.262899	-0.634940	2.524731
H	3.268776	0.454697	1.153335
O	4.230806	0.282906	1.013677
H	4.530143	-0.781593	2.433042
H	4.281473	-0.065408	0.117416
O	4.538049	-1.242096	3.300011
H	2.503392	-0.429454	3.327562
H	4.809501	-0.562481	3.922135

07.xyz

Fe	0.023201	0.000215	-0.156380
C	3.458270	0.068842	-0.371675
C	0.096580	-3.408395	-0.157088
C	-3.387329	-0.067622	0.246889
C	-0.038484	3.409569	-0.142852
N	1.495910	-1.392779	-0.238476
C	2.842339	-1.178325	-0.333914
C	3.547154	-2.440730	-0.403770
C	2.599011	-3.418635	-0.352152
C	1.318989	-2.748578	-0.248756
N	-1.378020	-1.450173	-0.005531
C	-1.149881	-2.798102	-0.035470
C	-2.397539	-3.518112	0.107522
C	-3.378286	-2.578612	0.233716
C	-2.726273	-1.289163	0.163073
N	-1.434371	1.393786	-0.003563
C	-2.775149	1.178990	0.165730
C	-3.477847	2.441299	0.241645
C	-2.535233	3.419378	0.119260
C	-1.260045	2.749686	-0.026811
N	1.439455	1.451130	-0.229825
C	1.209159	2.798924	-0.234601

C	2.461803	3.519296	-0.334512
C	3.447830	2.579813	-0.390307
C	2.793537	1.290245	-0.326406
H	4.547079	0.090702	-0.449980
H	0.117486	-4.500243	-0.168213
H	-4.470369	-0.089271	0.383990
H	-0.060814	4.501427	-0.149006
H	4.629292	-2.543868	-0.479082
H	2.733289	-4.499807	-0.375166
H	-2.489418	-4.603951	0.117953
H	-4.449468	-2.726169	0.368696
H	-4.553928	2.545606	0.378041
H	-2.669885	4.500682	0.134789
H	2.553316	4.605020	-0.352918
H	4.525024	2.725997	-0.465245
N	1.269391	-0.000396	2.482170
O	1.139935	-0.001798	3.868432
S	-0.027217	-0.006226	-2.428678
C	-1.763432	0.009973	-2.977118
H	-2.286995	0.912944	-2.629754
H	-1.770131	0.000176	-4.078930
H	-2.308574	-0.873855	-2.614091
H	0.147403	-0.004321	3.956127
N	0.130297	-0.002437	1.922242
O	-0.931278	-0.005626	2.657066

07_2W.xyz

Fe	-0.421056	-0.056898	-0.313273
C	2.724075	-0.872867	-1.458803
C	-1.094890	-3.362828	0.172451
C	-3.476880	0.769404	1.021651
C	0.291176	3.253048	-0.734071
N	0.616611	-1.777505	-0.591452
C	1.883444	-1.917895	-1.087341
C	2.237567	-3.319379	-1.169139
C	1.159949	-4.019319	-0.714153
C	0.152936	-3.042504	-0.355283
N	-1.986057	-1.088755	0.439125
C	-2.077767	-2.449923	0.547128
C	-3.349088	-2.818783	1.132115
C	-4.017391	-1.656169	1.380396
C	-3.153363	-0.581575	0.943487
N	-1.417037	1.667279	0.040788
C	-2.659819	1.811448	0.595594
C	-3.009855	3.212331	0.682562
C	-1.952284	3.909745	0.178297
C	-0.959544	2.931355	-0.212811
N	1.196175	0.981737	-0.966994
C	1.288258	2.342611	-1.074667
C	2.593431	2.715705	-1.579238
C	3.282323	1.553960	-1.776785
C	2.394153	0.477850	-1.394115
H	3.718931	-1.129993	-1.826229
H	-1.318342	-4.422041	0.315852
H	-4.446399	1.031394	1.449944
H	0.516191	4.312718	-0.871409
H	3.193952	-3.696811	-1.529343
H	1.040680	-5.097976	-0.617312
H	-3.667891	-3.842214	1.327807
H	-5.003422	-1.518806	1.823195

H	-3.945660	3.594062	1.089681
H	-1.831502	4.988401	0.082399
H	2.921729	3.739545	-1.755910
H	4.298168	1.417174	-2.145085
N	1.564151	-0.166277	1.831974
O	1.915041	0.069948	3.176532
S	-1.152994	-0.225395	-2.439253
C	-2.916062	0.223504	-2.504707
H	-3.079276	1.265947	-2.194475
H	-3.251272	0.109051	-3.547869
H	-3.523056	-0.434549	-1.865867
N	0.335168	0.083963	1.644925
O	-0.381737	0.473801	2.637807
H	4.416370	-1.294641	1.079201
O	5.279162	-0.906651	1.257233
H	3.784718	1.854571	1.419816
H	5.053131	-0.044847	1.648105
O	4.168404	1.596464	2.264632
H	1.034236	0.375035	3.536211
H	3.431816	1.149749	2.720817

08ts.xyz

Fe	0.032322	0.043085	-0.203432
C	3.466436	0.373499	-0.339640
C	0.357357	-3.334693	-0.389150
C	-3.376620	-0.304046	0.175026
C	-0.285467	3.416211	-0.045869
N	1.610961	-1.223680	-0.338242
C	2.941852	-0.914332	-0.383860
C	3.734554	-2.120426	-0.496356
C	2.855271	-3.161146	-0.523591
C	1.529673	-2.586437	-0.421801
N	-1.266868	-1.506153	-0.149657
C	-0.932161	-2.829362	-0.249201
C	-2.117765	-3.653272	-0.147052
C	-3.170178	-2.804388	0.029571
C	-2.623968	-1.464819	0.025608
N	-1.534202	1.300590	-0.010923
C	-2.856589	0.986534	0.150064
C	-3.645570	2.191955	0.285405
C	-2.773750	3.237166	0.203372
C	-1.455333	2.665454	0.028335
N	1.342725	1.584882	-0.194641
C	1.006244	2.908798	-0.141244
C	2.199337	3.729820	-0.170906
C	3.258826	2.875482	-0.238945
C	2.707389	1.536763	-0.257357
H	4.552254	0.479594	-0.382979
H	0.459805	-4.420027	-0.453078
H	-4.453991	-0.413421	0.314785
H	-0.390061	4.502546	-0.003056
H	4.822903	-2.145955	-0.542335
H	3.064555	-4.228130	-0.594238
H	-2.121392	-4.742023	-0.189939
H	-4.225071	-3.044530	0.159346
H	-4.725428	2.214617	0.429571
H	-2.982973	4.304626	0.268652
H	2.203952	4.818881	-0.133686
H	4.322624	3.109090	-0.272289
N	0.414297	-1.234402	3.265450

O	0.402222	-0.389868	4.409634
S	0.001087	0.156724	-2.441663
C	-1.716964	0.005282	-3.019948
H	-2.352401	0.808675	-2.620840
H	-1.689919	0.089413	-4.118576
H	-2.153882	-0.967364	-2.751801
H	0.270520	0.491604	3.980294
N	0.259754	-0.584948	2.202051
O	0.115108	0.719408	2.266991

09.xyz

Fe	0.011514	0.000110	-0.193105
C	3.441657	0.081811	-0.412347
C	0.089164	-3.405805	-0.220074
C	-3.400022	-0.081695	0.274085
C	-0.071420	3.406191	-0.196701
N	1.484223	-1.385758	-0.289699
C	2.829967	-1.167822	-0.379867
C	3.538014	-2.428678	-0.456668
C	2.591564	-3.408397	-0.417032
C	1.309504	-2.741452	-0.310314
N	-1.392425	-1.456354	-0.032611
C	-1.158715	-2.803018	-0.076739
C	-2.400309	-3.529936	0.086723
C	-3.382271	-2.596200	0.244343
C	-2.737175	-1.302433	0.167352
N	-1.459505	1.387587	-0.025488
C	-2.795476	1.169238	0.174604
C	-3.500774	2.430579	0.260882
C	-2.564039	3.410640	0.109601
C	-1.289711	2.744032	-0.059371
N	1.417244	1.454788	-0.278140
C	1.178923	2.800770	-0.289626
C	2.428257	3.527912	-0.391723
C	3.419725	2.594100	-0.438079
C	2.771851	1.300791	-0.369974
H	4.530617	0.107826	-0.486756
H	0.114610	-4.497454	-0.240694
H	-4.479485	-0.107671	0.437306
H	-0.097577	4.497948	-0.209549
H	4.620577	-2.529379	-0.528583
H	2.728279	-4.489073	-0.447352
H	-2.487920	-4.616170	0.090325
H	-4.449548	-2.750584	0.401405
H	-4.573955	2.533323	0.420000
H	-2.702633	4.491494	0.121306
H	2.514016	4.614005	-0.414876
H	4.496308	2.746134	-0.509584
N	1.242989	0.007595	2.417904
O	0.059226	-0.002040	1.783784
S	-0.067202	-0.006506	-2.467557
C	-1.810485	0.024252	-2.995807
H	-2.320996	0.937103	-2.654408
H	-1.828226	0.003370	-4.097415
H	-2.362826	-0.849888	-2.620211
N	1.186998	-0.000469	3.663403
O	-0.125905	-0.019180	4.197238
H	-0.677988	-0.020962	3.385357

10ts.xyz

Fe	-0.024685	0.093886	-0.172097
C	3.317518	0.862105	-0.419141
C	0.707302	-3.213062	-0.544703
C	-3.334172	-0.699435	0.361252
C	-0.759934	3.400637	0.183388
N	1.682648	-0.962802	-0.421731
C	2.958762	-0.478942	-0.506335
C	3.892376	-1.563469	-0.723885
C	3.154085	-2.707505	-0.775087
C	1.772448	-2.318154	-0.582144
N	-1.109737	-1.616525	-0.117431
C	-0.625535	-2.882050	-0.314313
C	-1.697792	-3.847673	-0.209862
C	-2.833566	-3.144138	0.065368
C	-2.453168	-1.749654	0.118607
N	-1.724674	1.141790	0.175763
C	-2.985424	0.648899	0.378500
C	-3.915382	1.733553	0.607125
C	-3.191064	2.887586	0.538369
C	-1.820501	2.502779	0.277544
N	1.071809	1.794437	-0.122724
C	0.578852	3.063161	0.006579
C	1.660401	4.025831	-0.038485
C	2.811011	3.312005	-0.192811
C	2.426937	1.917375	-0.247498
H	4.378222	1.106423	-0.503278
H	0.941014	-4.271154	-0.678496
H	-4.381158	-0.951548	0.541374
H	-0.996575	4.462194	0.282146
H	4.971287	-1.443878	-0.817019
H	3.495085	-3.732440	-0.917559
H	-1.574786	-4.924747	-0.318981
H	-3.844129	-3.518632	0.225835
H	-4.980892	1.608561	0.798225
H	-3.534097	3.914244	0.663995
H	1.534690	5.104725	0.048468
H	3.835428	3.676647	-0.261389
N	1.379399	0.086248	2.544950
O	0.133100	-0.072682	1.843041
S	-0.194042	0.322230	-2.406535
C	-1.885935	-0.118470	-2.917933
H	-2.634541	0.536360	-2.448260
H	-1.944176	0.007700	-4.010925
H	-2.124103	-1.163433	-2.671116
N	1.468166	-0.833337	3.401941
O	0.452263	-1.685157	3.411046
H	-0.114277	-1.001974	2.400776

11.xyz

Fe	-0.051817	0.095161	-0.174966
C	3.153550	1.302539	-0.457188
C	1.108691	-3.083238	-0.578873
C	-3.216394	-1.135761	0.409648
C	-1.216846	3.272869	0.212198
N	1.776153	-0.723875	-0.453215
C	2.975008	-0.073394	-0.551877
C	4.041305	-1.022031	-0.792529
C	3.461628	-2.253939	-0.843070
C	2.043738	-2.053923	-0.626375
N	-0.896443	-1.744739	-0.106969

C	-0.251652	-2.933939	-0.322564
C	-1.183631	-4.033932	-0.208416
C	-2.398280	-3.489869	0.090682
C	-2.206873	-2.057730	0.148445
N	-1.869867	0.904865	0.209981
C	-3.050928	0.247146	0.427269
C	-4.114412	1.196996	0.670781
C	-3.552325	2.437889	0.595577
C	-2.146262	2.240938	0.316406
N	0.807985	1.925724	-0.124616
C	0.152532	3.117405	0.017994
C	1.095926	4.215332	-0.033103
C	2.329148	3.661488	-0.204974
C	2.133173	2.228647	-0.264942
H	4.171106	1.686064	-0.552948
H	1.479041	-4.099922	-0.724254
H	-4.217450	-1.526267	0.603359
H	-1.592080	4.292966	0.318260
H	5.093084	-0.758921	-0.899298
H	3.934182	-3.223342	-0.997808
H	-0.919964	-5.084308	-0.327555
H	-3.346693	-3.997045	0.265322
H	-5.150994	0.930259	0.874991
H	-4.028460	3.409095	0.728043
H	0.829244	5.267476	0.062669
H	3.295202	4.159199	-0.282668
N	1.455543	0.253624	2.560305
O	0.170265	-0.042728	1.855868
S	-0.287077	0.312922	-2.399270
C	-1.901788	-0.371131	-2.890278
H	-2.731396	0.162956	-2.404144
H	-1.994540	-0.247973	-3.981193
H	-1.977807	-1.441589	-2.648693
N	1.716063	-0.690905	3.343536
O	0.906079	-1.706973	3.387594
H	0.029337	-0.958997	2.263620

12ts.xyz

Fe	-0.057583	0.108866	-0.174216
C	2.999261	1.662883	-0.430513
C	1.438503	-2.913539	-0.655463
C	-3.078327	-1.468493	0.339262
C	-1.554271	3.130462	0.308657
N	1.848287	-0.499310	-0.480241
C	2.969000	0.278662	-0.563579
C	4.131556	-0.543664	-0.824444
C	3.688756	-1.829917	-0.899165
C	2.257370	-1.788480	-0.679192
N	-0.702693	-1.811185	-0.169404
C	0.068530	-2.917931	-0.406685
C	-0.740466	-4.114652	-0.330441
C	-2.009603	-3.712413	-0.034020
C	-1.973960	-2.269686	0.062423
N	-1.954424	0.708051	0.221237
C	-3.060651	-0.077091	0.404370
C	-4.219565	0.746891	0.671374
C	-3.790238	2.041609	0.645404
C	-2.369590	2.003112	0.371452
N	0.602877	2.020946	-0.072540
C	-0.176227	3.129419	0.111107

C	0.643341	4.323939	0.093562
C	1.928023	3.912150	-0.100549
C	1.886721	2.468780	-0.206544
H	3.969202	2.156318	-0.518068
H	1.916825	-3.880992	-0.820621
H	-4.033317	-1.969245	0.510762
H	-2.036252	4.100496	0.448244
H	5.148309	-0.165725	-0.926474
H	4.263037	-2.739100	-1.074201
H	-0.363972	-5.127065	-0.472966
H	-2.899494	-4.322972	0.115859
H	-5.223598	0.365890	0.856157
H	-4.366506	2.952253	0.807084
H	0.265514	5.337755	0.223396
H	2.834018	4.513828	-0.166196
N	1.640006	0.084676	2.616752
O	0.177137	-0.014897	1.822223
S	-0.308846	0.367099	-2.402644
C	-1.858287	-0.436557	-2.922891
H	-2.734837	0.030222	-2.449376
H	-1.943681	-0.317418	-4.014895
H	-1.856730	-1.510651	-2.685634
N	1.729681	-0.860255	3.377975
O	0.869859	-1.798149	3.523226
H	-0.101199	-0.876697	2.194024

13.xyz

Fe	0.136594	0.003082	-0.188280
C	-3.171926	0.095234	-1.132057
C	0.223053	3.406860	-0.161549
C	3.412073	-0.088909	0.887136
C	0.053440	-3.399991	-0.229135
N	-1.218349	1.465290	-0.582729
C	-2.523705	1.314989	-0.948574
C	-3.147301	2.612395	-1.126868
C	-2.187472	3.542710	-0.860912
C	-0.983909	2.808302	-0.517373
N	1.556427	1.386157	0.259357
C	1.393157	2.742421	0.203150
C	2.616638	3.407935	0.602890
C	3.514077	2.426879	0.909193
C	2.836299	1.165567	0.689317
N	1.486457	-1.456911	0.228897
C	2.775285	-1.309021	0.662684
C	3.389412	-2.606715	0.856689
C	2.444053	-3.535514	0.532205
C	1.255401	-2.801987	0.146752
N	-1.289457	-1.381326	-0.610250
C	-1.122334	-2.735444	-0.571868
C	-2.360912	-3.401802	-0.929397
C	-3.272768	-2.419561	-1.176890
C	-2.585266	-1.158744	-0.973015
H	-4.222842	0.124186	-1.427914
H	0.252252	4.498964	-0.156415
H	4.446519	-0.118073	1.236599
H	0.028259	-4.492096	-0.245197
H	-4.185885	2.768812	-1.417827
H	-2.267417	4.629478	-0.884946
H	2.751649	4.488772	0.643028
H	4.543525	2.528993	1.252402

H	4.412416	-2.766974	1.196925
H	2.524675	-4.622346	0.551137
H	-2.495313	-4.482488	-0.974856
H	-4.317595	-2.517853	-1.470913
N	-3.366210	-0.010976	2.727489
O	-0.448200	-0.000684	1.605573
S	0.679201	0.008133	-2.458505
C	2.495340	-0.016342	-2.626469
H	2.934027	-0.916414	-2.168257
H	2.745263	-0.017513	-3.700181
H	2.959047	0.869119	-2.164386
N	-2.558802	-0.024103	3.502770
O	-1.733001	-0.038498	4.357691
H	0.336100	-0.013850	2.161850
